# Blurring of ancient wall paintings caused by binder decay in the pigment layer

**DOI:** 10.1038/s41598-020-78117-4

**Published:** 2020-12-03

**Authors:** Lizhen Zheng, Zhuorui Wang, Shukun Shen, Yin Xia, Yuhu Li, Daodao Hu

**Affiliations:** 1 Engineering Research Center of Historical and Cultural Heritage Protection, Ministry of Education, School of Materials Science and Engineering, Shaanxi Normal University, Xi’an, 710062 China; 2 Emperor Qin Shihuang’s Mausoleum Site Museum, Lingtong, Xi’an, 710600 China

**Keywords:** Chemistry, Materials chemistry, Physical chemistry, Surface chemistry

## Abstract

In this paper, the effect of binder decay rather than a change in the pigments on the blurring of ancient wall paintings was researched. The simulated wall paintings were prepared by brushing an aqueous solution containing gelatine and ochre grains on the surface of cylindrical compressed soil samples. Then, the dried samples were calcined at 650 °C for 2 h to obtain the simulated wall paintings with the degraded binder gelatine. Next, the calcined samples were brushed with a certain amount of acetone solution containing an ionic liquid ([BMIm]PF_6_) to obtain the corresponding repaired samples. Based on the results from various characterization methods (UV–vis, FTIR, XRD, XPS, SEM, TG), the following conclusions were drawn. The degradation of the binder caused by calcination increases the surface roughness of the painting layer, resulting in enhanced scattering. In this case, because scattering decrease the light absorption by the pigments, even if unchanged pigment exists in the painting layer, its colour can become blurred. The filling of the ionic liquid into the pores caused by gelatine decay in the painting layer can decrease the scattering, and the blurred colour can be restored to some extent. As typical examples, this principle was successfully applied to restore the blurred colour of an ancient Chinese wall painting (Tang Dynasty) and a pottery (Eastern Han Dynasty).

## Introduction

**Figure 1:**
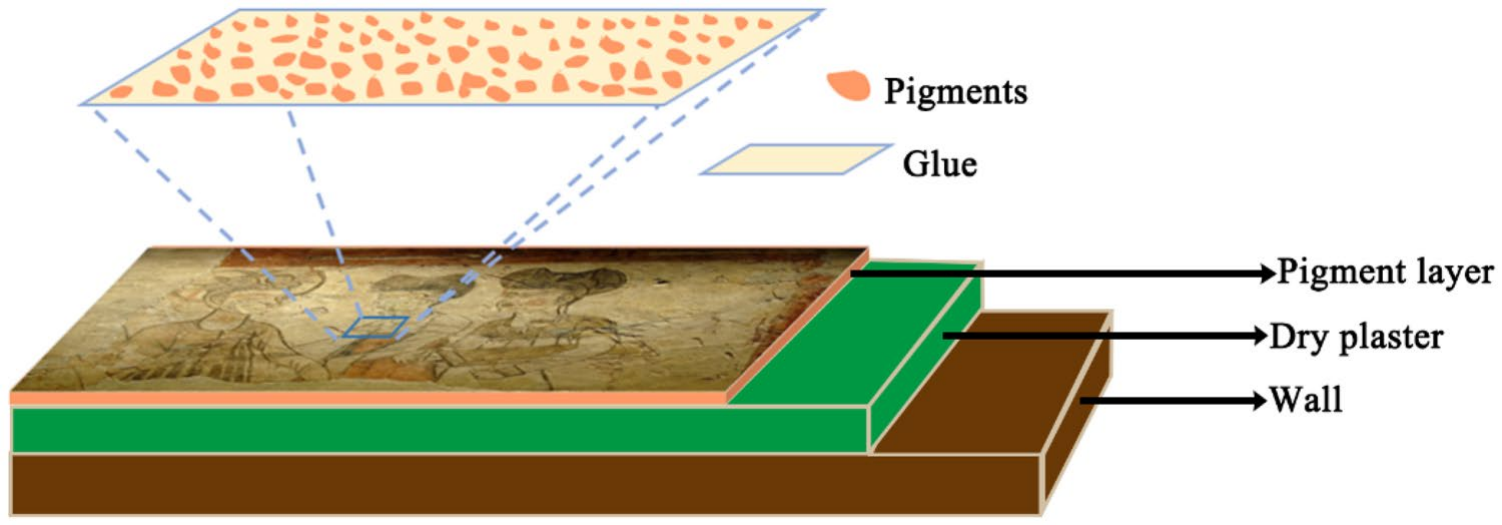
Stratigraphy of a typical secco.

Wall paintings are a type of art painted on the walls around caves, buildings, and burial chambers, which can be traced back to more than sixty thousand years ago^1^. Although the workmanship of the wall paintings changes with time, there is no substantial change in the basic structure. In simple terms, wall paintings refer to the pictures painted on a wall carrier. Wall carriers are divided into natural rock, brick, and clay. Because pigment grains are combined in a painting layer, wall painting techniques are classically divided into *buon* and *secco*. For the *buon* technique, pigments (dispersed in lime water) are directly applied on the wet lime plaster layer, and upon carbonation, they are embedded into the crystalline carbonate network, forming a smooth surface. For the *secco* technique, pigments (dispersed in gum or animal glue) are applied on the dried plaster layer and dispersed to the binder layer. Worldwide, there are obvious regionalities in wall painting techniques. In ancient china, the so-called *secco* technique was popularly used. A typical structure of a wall painting in ancient China is shown in Fig. 1. The writing brush was dipped a mixture of pigment granules, and glue was used to depict the desired picture. Unfortunately, changes in some environmental factors have caused the material in the coating layer to decay, seriously affecting the artistic quality of the wall painting. The blurring of a painting is one of the most common and typical phenomena, and is universally attributed to changes in the pigments^2^. This conclusion is mainly based on the principle that the colour of the wall painting is only dependent on the pigments. Namely, the blurring of painting is only ascribed only to the change in the pigments in wall painting.

Undeniably, there is a probability of changes in pigments in ancient wall paintings. As reported in the literature^2^, although centuries of practical knowledge have allowed artists and artisans to select mineral pigments that were not susceptible to fading, the change in pigments used in ancient wall painting may still occur for the following reasons. First, artists and artisans are constantly experimenting, and using unstable pigment is possible according to their savoir-faire and practical considerations^3,4^. Additionally, pigments are subjected to varying environmental conditions, including environmental pollution and seasonal climatic changes. Finally, some unsuitable conservation treatments might alter the stability of pigment in paint layers. Nevertheless, it is illogical that the blurring of all colours in an ancient wall painting is attributed to the instability of the pigments used.

In fact, in addition to the pigment, the surface roughness is also an important factor for the clarity of the image^5^. Light scattering caused by rough surfaces of the wall painting can decrease the absorption of light by the pigments located in the wall painting. As a result, the blurring of the pigment colour is inevitable. It could be incomplete and sometimes even incorrect to attribute the colour blurring of the wall painting only to the changes in pigments.

**Figure 2:**
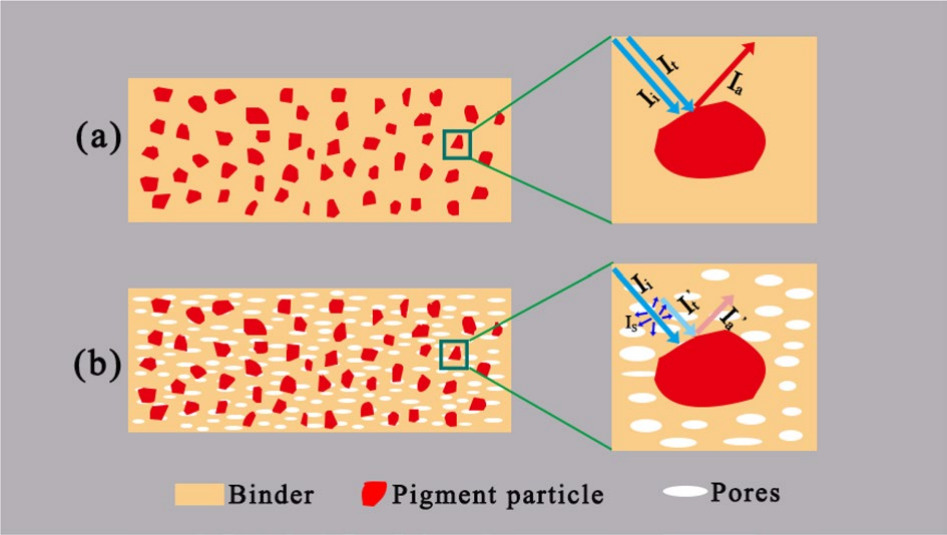
Optical model based on the Lambert–Beer model of layered surface objects. **(a)** Fresh wall painting; **(b)** wall painting with a degraded binder layer.

Figure 2 schematically shows the optical model based on the Lambert–Beer model of a wall painting layer. For sake of better understanding, a local area around a pigment grains is used as a model to illustrate the effect of the binder on the absorbance of the pigment. For a given strength of incident light (I_i_), the absorbance of the pigment (I_a_) depends on not only the pigments but also the transmittance of the binder around pigment the grains (I_t_). For an absence of pores in the binder phase, as shown in Fig. 2a, there is almost no loss of the incident light that reaches the surface of the pigment particles through the binder. In this case, the absorbance of the pigment (I_a_) is relatively strong. However, for the presence of pores in the binder phase, as shown in Fig. 2a,b strong scattering (I_s_) caused by the pores in the binder phase causes the incident light that reaches the surface of the pigment particles through the binder significantly decrease. As a result, the observed colour of the pigment lightens in this case even if the pigment does not change.

The effects of pores on optical haze, which is practically used for evaluating transparency, can be precisely studied with Mie scattering theory. The transmission can be expressed by the Lambert Beer equation: T = (1 − R)^2^ exp(-*C*_sca_*t*)^6–8^. Here, R is the reflectivity, C_sca_ [m^−1^] is the effective scattering coefficient, and *t* is the sample thickness. R can be calculated using the values of the refractive index of the media (n_med_) and the discrete phase (n_dis_).

1 $$ {\text{R }} = \left( {{\text{n}}_{{{\text{med}}}} - {\text{ n}}_{{{\text{dis}}}} } \right)^{{2}} /\left( {{\text{n}}_{{{\text{med}}}} + {\text{ n}}_{{{\text{dis}}}} } \right)^{{2}} $$

Obviously, for a fresh painting, the binder is the media, and the pigments are the discrete phases. For painting with binder degradation, both pores and pigment are the discrete phases. Because the refractive index of air in the pores is lower than that of the pigments, the R of the wall painting with binder degradation is higher than that of the fresh wall painting. As a result, the presence of pores in the painting layer decreases the intensity of the incident light irradiated on the pigments, lightening the colour of pigments. For C_sca_, its value changes with wavelength and the distribution of the dispersing phases.

2 $$ C_{{{\text{sca}}}} = {\text{N}}_{{{\text{dis}}}} \cdot{\text{G}}_{{{\text{dis}}}} \cdot{\text{ Q}}_{{{\text{sca}}}} $$

where N_dis_ is the disperse density, G_pore_ is the geometrical cross section of the disperses, and Q_sca_ is the scattering efficiency with a change in the wavelength. This equation indicates the change in the scattering of the dispersed phase size as a function of the light wavelength. The details of the calculations are described elsewhere^9^. Nonetheless, it is related to the hue of the scattered light. However, all colours on the ancient wall paintings often weaken rather than exhibit a change in hue. This case matches the Mie scattering that occurs when the size of the dispersed phase is comparable to the wavelength of the incident light and the wavelength of diffuse reflection is similar to that of the incident light. As shown in Fig. 2b, if the pores in the binder phase possess the sizes mentioned above, then the binder phase can mostly completely diffusely reflect the incident light. As a result, the binder phase in this case actually becomes an optical haze layer^10,11^, making the absorbance of the pigments obviously decrease. Additionally, the scattering interface can exist not only at air voids inside the coating but also at the surface between the coating and ambient air. For fresh wall paintings, the resultant surface is flat enough to ignore the influence of surface scattering^8^. However, this case is very obvious in the blurring of a wall painting because the decay always starts at surface. To a great extent, this effect cannot be ignored in the colour blurring of ancient wall paintings in China. If the air in the pores of a wall painting is replaced by a substance with a higher reflective index to decrease the light scattering^7,12,13^, then the blurred wall painting can be recovered.

In China, ancient wall paintings are mainly available in burial chambers, caves, temples, and palaces, and these wall paintings are blurred to varying degrees, and some are barely recognizable. Generally, almost all ancient wall paintings in China were prepared by using the *secco* technique, and the materials used in the painting layer are a mixture of inorganic mineral pigments and organic glue^2,14–16^. The inorganic mineral pigments used are commonly stable under natural conditions. Therefore, it is not reasonable that the blurring of ancient Chinese murals is absolutely attributed to the changes in pigments. Based on the previous statement, we presume that the blurring of ancient Chinese wall painting is largely related to the degradation of the binder rather than the variation in pigments. The binders in ancient Chinese wall painting are organic materials (animal glue or vegetable glue), and these materials are prone to degradation^15–17^. The degradation of the binder leads to the formation of numerous pores in the painting layer, as shown in Fig. 2b, which enhances the scattering strength of the painting layer. In this case, the scattering leads to the blurring of the colour of the pigments.

To verify our conjecture that the formation of pores caused by the degradation of the binder plays an indispensable role in the blurring of the wall painting, we present a model experiment to simulate the blurring of the wall painting caused by some pores in the painting layer. In this model experiment, the formation of some pores in the painting layer was carried out through the calcination of the painting layer containing an organic binder and stable pigments. Additionally, filling the pores with a trace amounts of an ionic liquid as an example for the restoration of the blurred colour of pigments was also performed. To the best of our knowledge, such a simulation investigation is the first of its kind. Our investigation could be of theoretical and practical significance in deepening the understanding of the blurring mechanism of wall paintings and guiding restoration.

## Results and discussion

### Effect of calcination on red ochre

**Figure 3:**
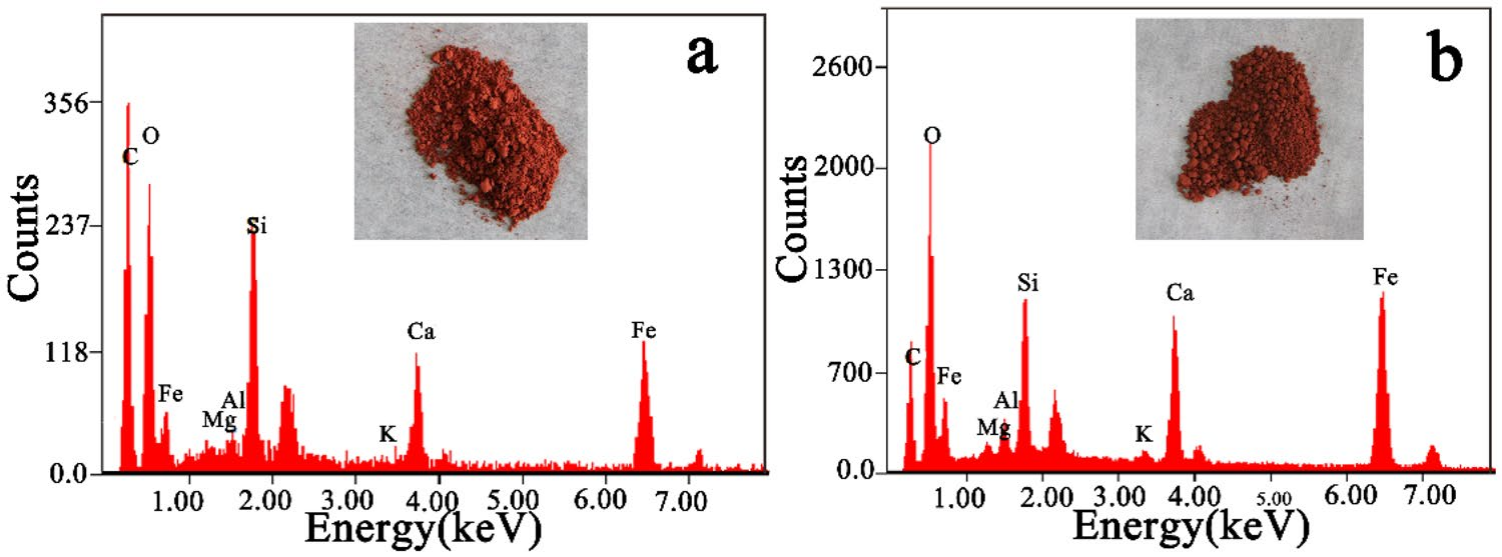
EDS spectra of the red ochre pigment before **(a)** and **(b)** after calcination. The insets in **(a)** and **(b)** are the corresponding photographs.

**Figure 4:**
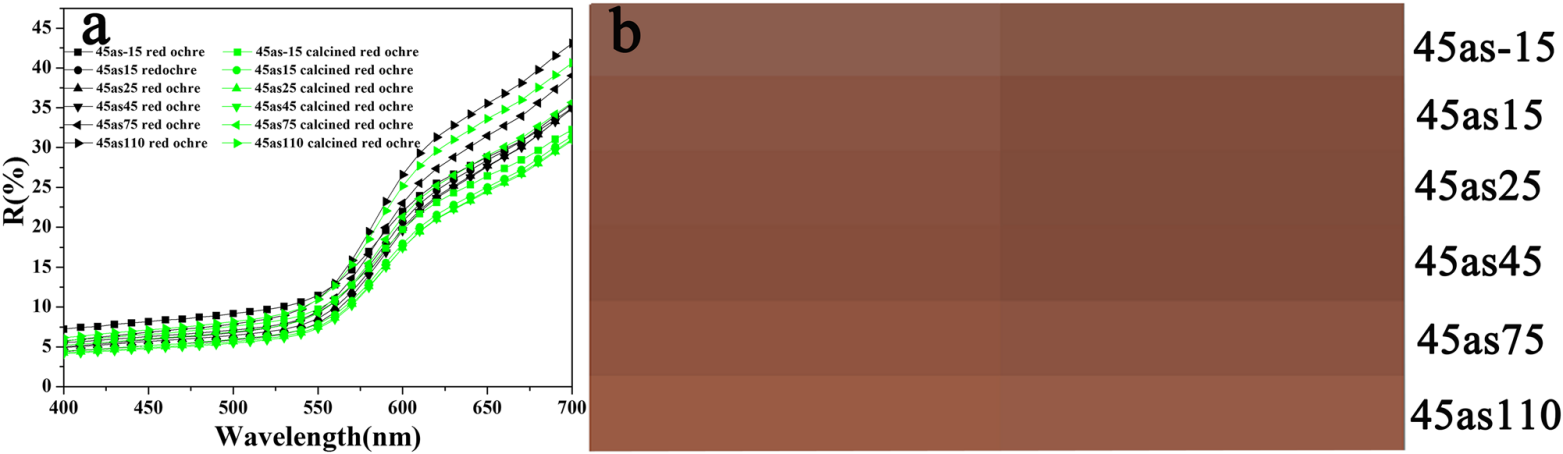
**(a)** Multiangle reflectance spectra of red ochre (black curves) and calcined red ochre (green curves). **(b)** Colour images of the red ochre (left) and calcined red ochre (right) at different viewing directions.

The simulation of a wall painting with binder decomposition can be obtained through the calcination of the wall paintings containing gelatine and red ochre. The red ochre selected in the simulated experiment is considered for its stability at high temperatures. To confirm that red ochre was unchanged after calcination, the component of red ochre before and after calcination was determined. From Fig. 3 (insets), it can be seen that the colour of red ochre after calcination was slightly darker than that of the original ochre pigment. This funding was confirmed by the reflectance spectra and colorimetric parameters, as shown in Fig. 4 and Table 1. Compared with pristine red ochre, the multiangle reflectance spectra of calcined red ochre are slightly reduced (Fig. 4a), and the colour detected from different viewing directions is darker (Fig. 4b). Table 1 indicates that although the L^*^ and C^*^ values for the calcined red ochre decrease slightly, the h* value almost does not change, implying that the hue of the calcined red ochre has not changed. This result at least shows that the calcination does not cause the red ochre to become lighter. This result does not contradict the verification of the proposed blurring model of the wall paintings.

**Table 1:** The L^*^C^*^h^*^ of the simulated wall paintings (2S, 4S), the calcined simulated wall paintings (F-2S, F-4S), and the calcined simulated wall treated with the ionic liquid (CF-2S, F-4S), red ochre and calcined red ochre.

Samples	L*	C*	h*
2S	51.19 ± 0.03	34.34 ± 0.03	53.78 ± 0.03
F-2S	57.52 ± 0.03	29.69 ± 0.03	53.81 ± 0.03
CF-2S	46.72 ± 0.03	29.19 ± 0.03	54.62 ± 0.03
4S	42.70 ± 0.03	33.02 ± 0.03	49.77 ± 0.03
F-4S	56.91 ± 0.03	26.15 ± 0.03	52.03 ± 0.03
CF-4S	44.22 ± 0.03	29.68 ± 0.03	49.92 ± 0.03
Red ochre	39.72 ± 0.03	31.75 ± 0.03	42.87 ± 0.03
Calcined ochre	37.88 ± 0.03	29.32 ± 0.03	42.75 ± 0.03

Note: L* = brightness, C* = saturation of colour, h* = hue of colour.

The 2S and 4S refer to the paintings prepared by brushing the mixture of gelatine and ochre for two and four times; The F-2S and F-4S refer to the samples (2S and 4S) after calcination; The CF-2S and CF-4S refer to the samples (F-2S and F-4S) treated by the ionic liquid.

**Figure 5:**
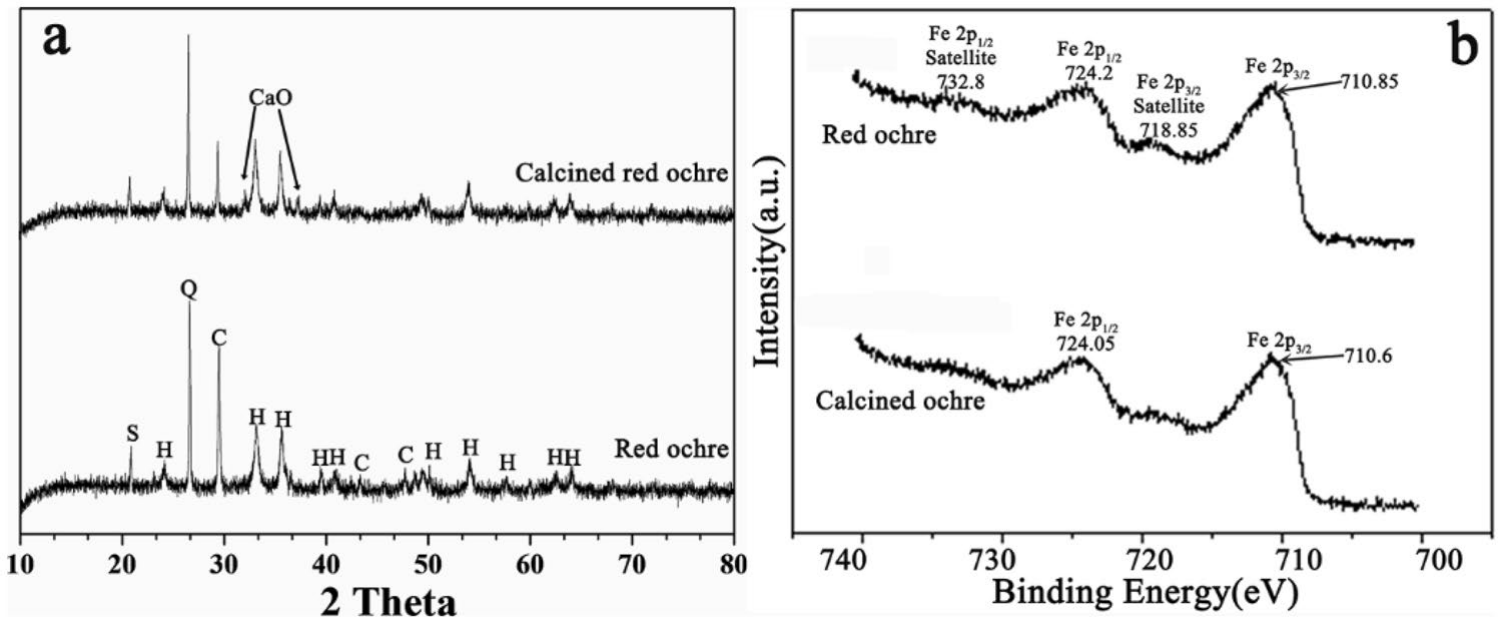
**(a)** XRD patterns of red ochre and calcined red ochre. Mineral phases: (S) silico-aluminate, (C) calcite, (H) haematite and (Q) quartz. **(b)** High resolution XPS spectra of Fe 2p in the ochre pigment before and after calcination.

Additionally, the components of red ochre before and after calcination were also analysed using EDS, XRD, and XPS. The corresponding EDS spectra are shown in Fig. 3. The results indicated that the spectra of red ochre before and after calcination are almost the same except for a slight decrease in the carbon content. The decrease in the carbon content is possibly related to the decomposition of carbonate in the red ochre pigment^18^. Figure 5a shows the XRD spectra for the red ochre before and after calcination. The characteristic peaks including haematite^19^, calcite^20^, quartz^21^, and Al2Si2O5(OH)4^22^ exist in the XRD spectra. After calcination, the characteristic peaks of α-Fe_2_O_3_ do not change^23^. This conclusion is also supported by the XPS spectra shown in Fig. 5b. The spectra related to Fe2p_1/2_ in the original red ochre are almost the same as those in the calcined red ochre. However, the characteristic peaks related to white calcite decreases obviously (in Fig. 5a), indicating the decomposition of carbonate in the ochre pigment. This change may be one of the reasons for the deepening colour of the ochre pigment. Probably, another reason is attributed to the increase in the grain size of pigment caused by the calcination.

According to the characterization results mentioned above, it can be concluded that calcination does not cause colour blurring and obvious changes in the components of red ochre.

### Effect of calcination on gelatine

**Figure 6:**
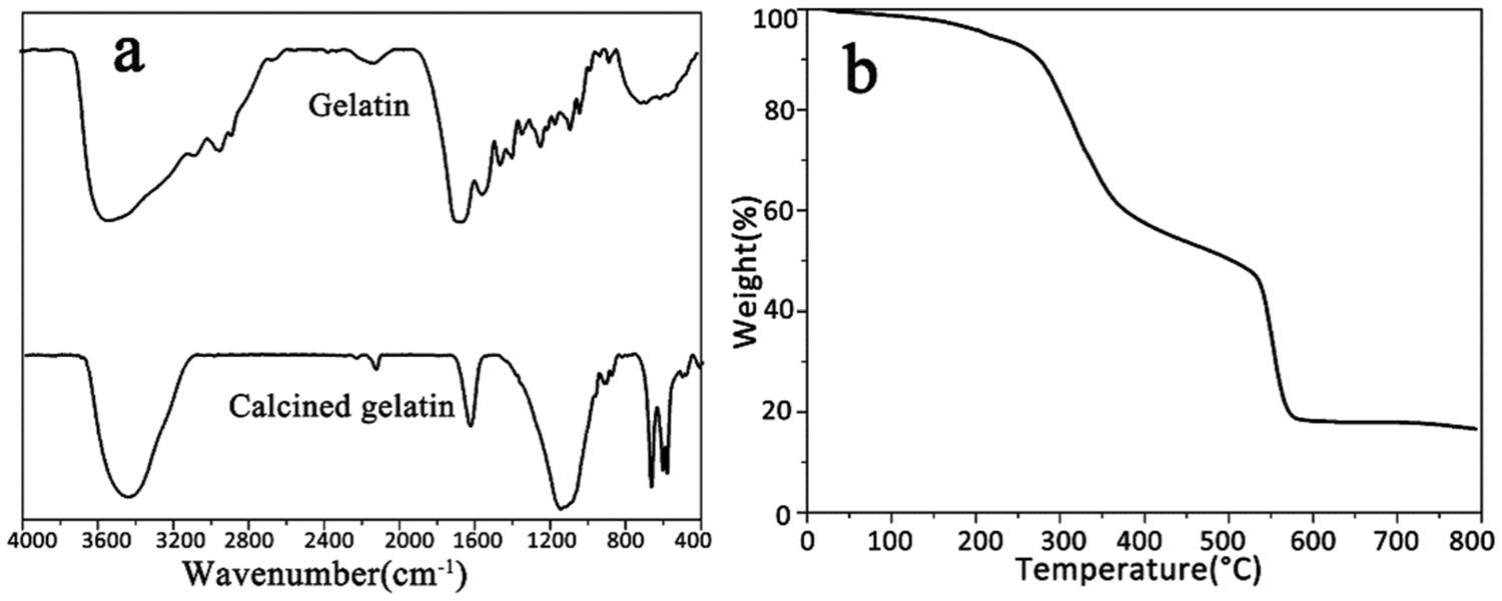
FTIR spectra of gelatine before and after calcination **(a)** and TG curve of gelatine **(b)**.

**Figure 7:**
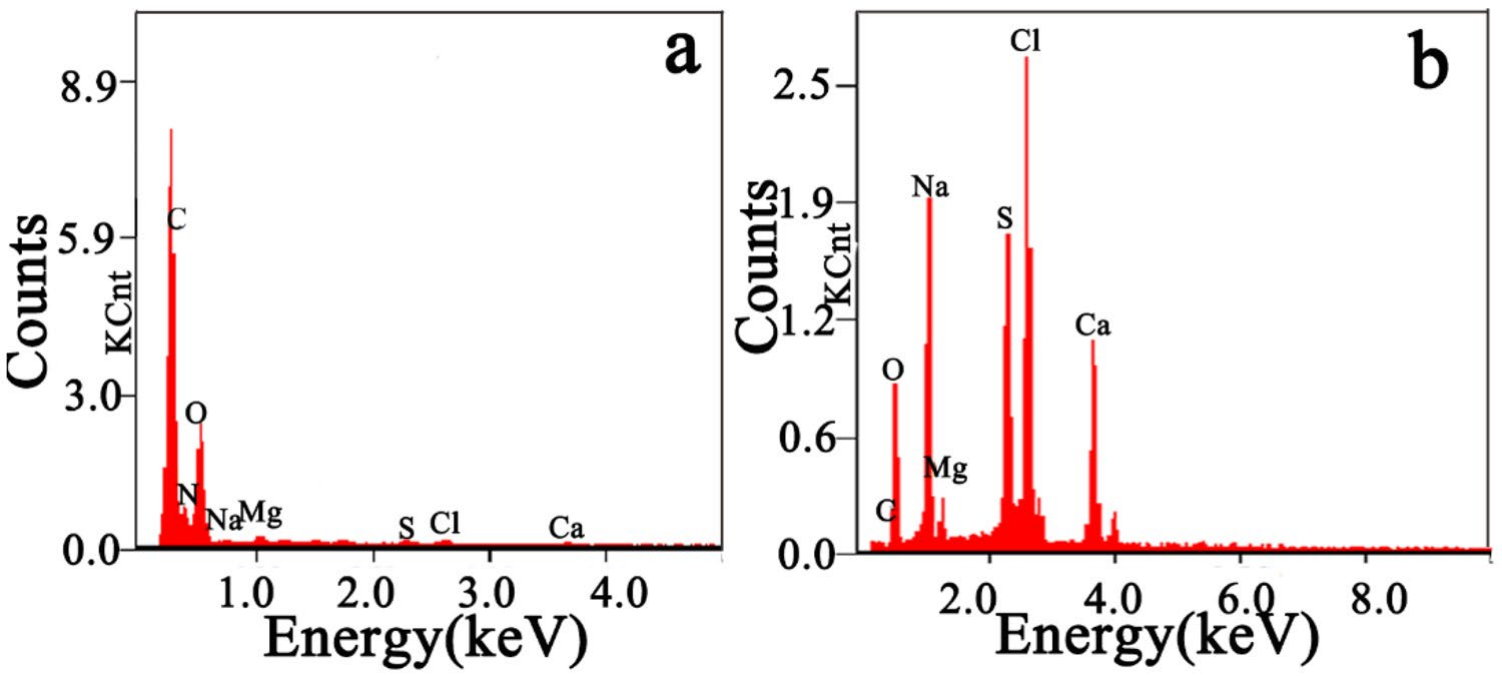
EDS spectra of gelatine before **(a)** and after calcination **(b)**.

The gelatine used as a binder in the simulated wall painting was analysed before and after calcination. The FTIR spectra of gelatine before and after calcination are shown in Fig. 6a. Compared with the original gelatine, the peaks for the calcined gelatine at 3552, 3072, 1660, 1544, and 1238 cm^−1^ attributed to amid A, amid B, amid I, amid II, and amid III disappear, respectively^24,25^, indicating that gelatine was degraded during calcination, and the new peaks are related to the residual inorganic matter after calcination, possibly derived from the additives^26^ and impurities in the gelatin. The TG curve of gelatine shown in Fig. 6b indicates that the gelatine had onset decomposition temperatures of 270–272 °C and is decomposed above 600 °C. The residue, is related to the presence of inorganic impurities in the gelatine. The peaks at 603 cm^−1^ and 567 cm^−1^ assigned to the asymmetric bending vibration of the PO_4_^3−^ group, and the peaks at 1092 cm^−1^ related to the antisymmetry stretching vibration of the PO_4_^3^ group are found, implying that phosphate exists in the residue of the calcined gelatine^27,28^. These results are consistent with those in the literature^29^. The EDS spectra of gelatine before and after the calcination of gelatine shown in Fig. 7 further verify this conclusion. Compared with the original gelatine, the calcination markedly decrease the carbon content and significantly increases the content of metal elements. In fact, the residual inorganic matter in calcined gelatine is caused by additives during the preparation of the gelatine^30^. The above results show that gelatine can completely degrade when it is heated above 600 °C.

### Effect of calcination on the mixture of red calcined ochre and gelatine

**Figure 8:**
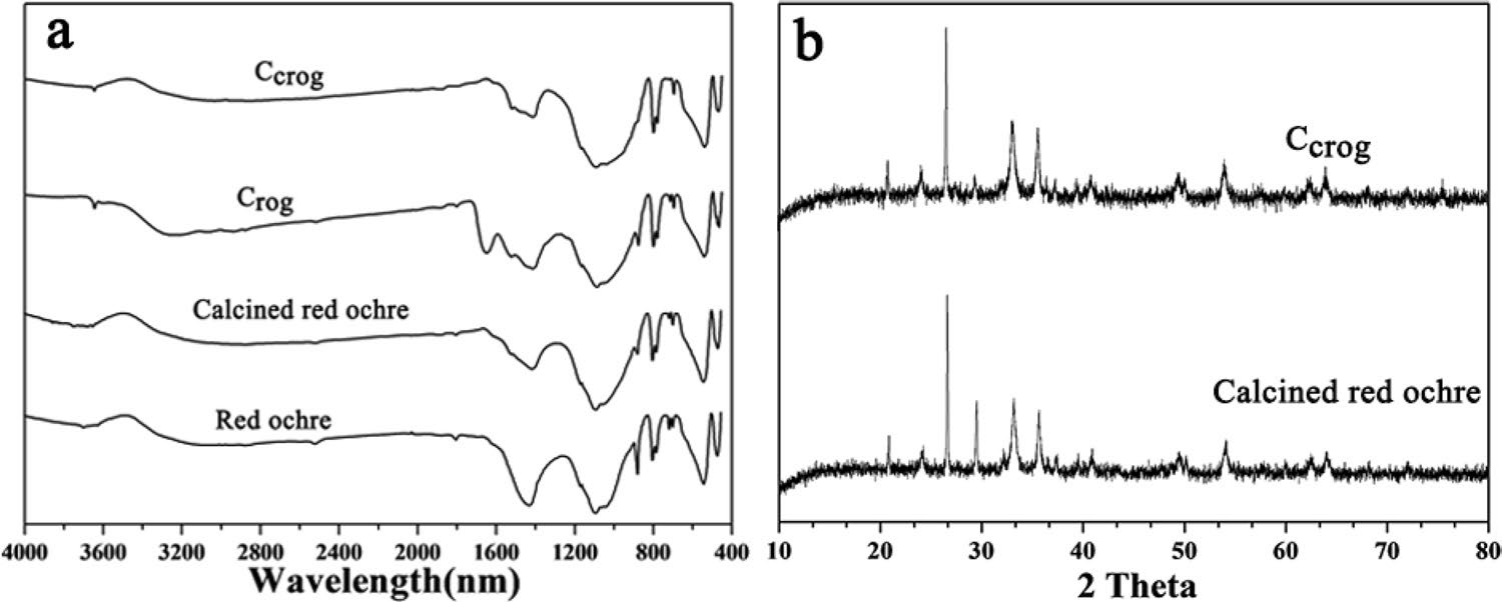
**(a)** FTIR spectra of red ochre, calcined red ochre, C_rog_ (the mixture of calcined ochre and gelatin solution) and C_crog_ (C_rog_ after calcination), **(b)** XRD of calcined red ochre and C_crog_.

To confirm the decomposition of the gelatine binder and the stability of red calcined ochre in the painting layer by calcination treatment, the the FTIR spectra of the painting layer containing red calcined ochre and gelatine after calcination (C_crog_) and red ochre were acquired, and the corresponding results are presented in Fig. 8a. The following results can be found. For red ochre, the characteristic peaks at 462 and 538 cm^−1^ (attributed to α-Fe_2_O_3_^31^) before and after calcination are the same. These indicate that the calcination does not affect the composition in the red ochre. Additionally, the decrease in the peak at 1432 cm^−1^ (the vibrations in ionic carbonate) is related to the decomposition of CaCO_3_. It appears at different wavenumbers, including at 1432 cm^−1^ (the vibrations in ionic carbonate), at 1030 cm^−1^ (asymmetric stretching of Si–O–Si), at 913 cm^−1^ (Si–O stretching band), and at 795 cm^−1^ (vibrations of Si–O–Al^22^). The above sets of peaks are due to the presence of calcite^20^, quartz^21^, and Al_2_Si_2_O_5_(OH)_4_^22^ in the red ochre. These results agree with the XRD results shown in Fig. 5. For the sample C_crog_, the characteristic peak at 1660 cm^−1^ attributed to amid I disappears, indicating the decomposition of gelatin. Compared with Fig. 6, the faint change at 462 and 538 cm^−1^ emerge, and the peaks related to phosphate appear, showing that the painting layer contains trace red ochre and that phosphate originates from the residue of the calcined binder. To further confirm the existence of red ochre in C_crog_, XDR spectra of C_crog_ and calcined red ochre are detected, as shown in Fig. 8b. The result clearly verifies that red ochre in C_crog_ remains.

These results indicate that calcination could degrade the binder gelatine in the painting layer without affecting the red ochre pigment. In other words, employing calcination to construct the model of the painting layer with degraded gelatine and unchanged red ochre is feasible.

### Surface morphology of the simulated wall samples

**Figure 9:**
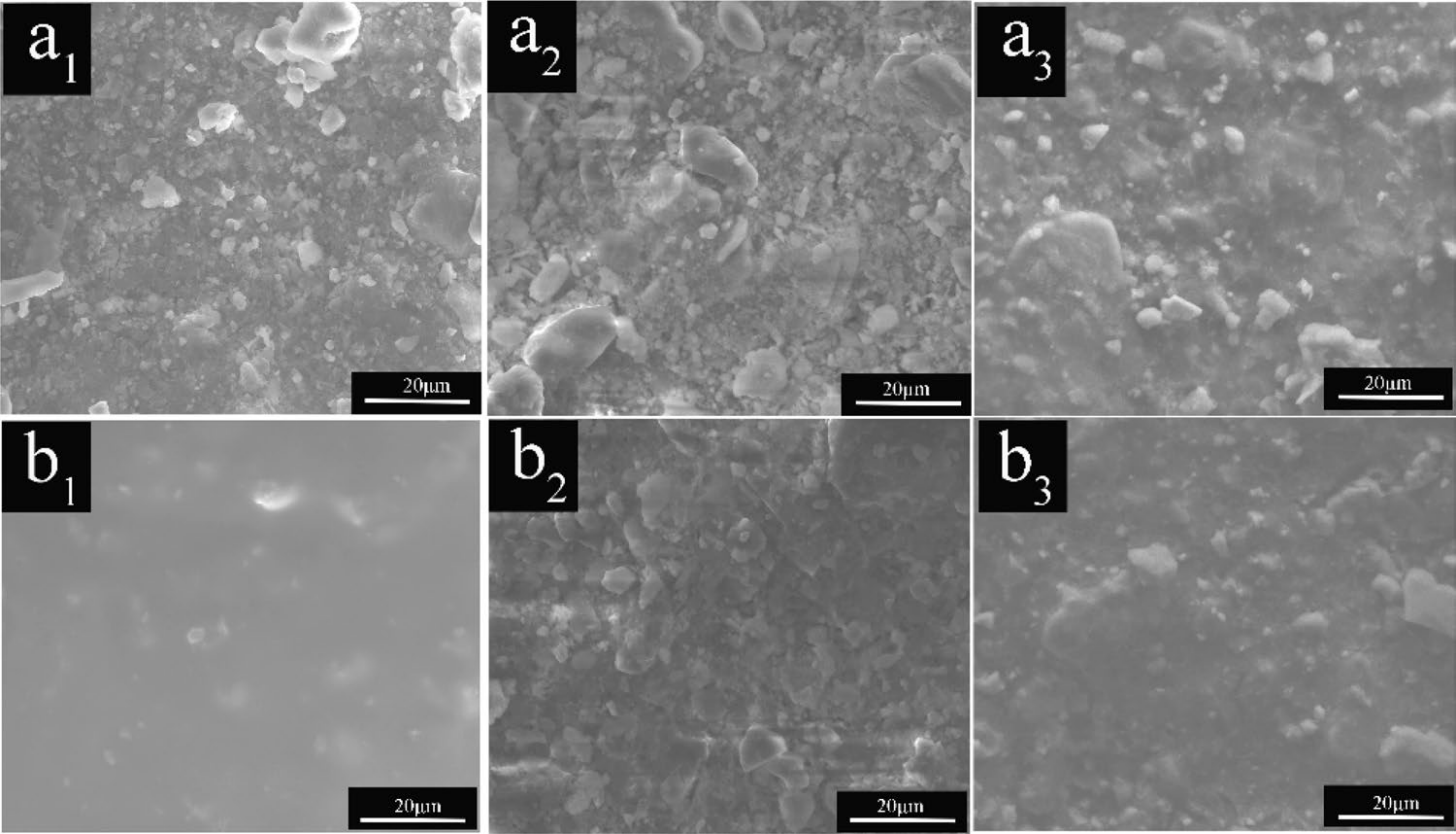
SEM images of **(a**_**1**_**,b**_**1**_**)** 2S and 4S, **(a**_**2**_**,b**_**2**_**)** F-2S and F-4S and **(a**_**3**_**,b**_**3**_**)** CF-2S and CF-4S.

**Figure 10:**
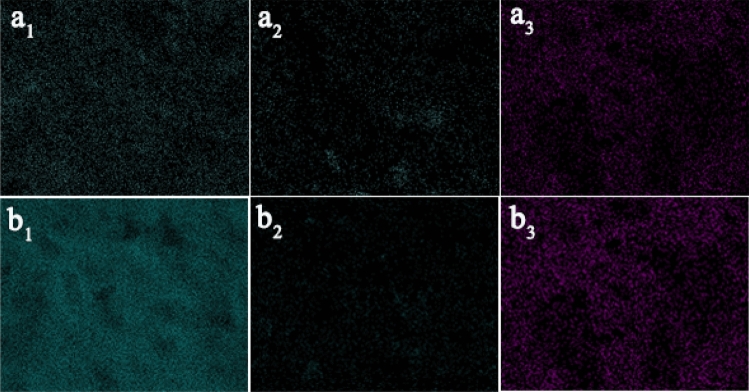
Carbon mapping of **(a**_**1**_**,b**_**1**_**)** for 2S and 4S and **(a**_**1**_**,b**_**1**_**)** for F-2S and F-4S. Fluorine mapping of **(a**_**3**_**)** for CF-2S and **(b**_**3**_**)** for CF-4S.

The simulated wall paintings coated with a mixture of gelatine and red calcined ochre for different brush numbers are denoted as 2S and 4S, respectively. The calcined corresponding samples are represented as F-2S and F-4S, respectively. The SEM images of the aforementioned samples are shown in Fig. 9. Before calcination, with an increase in the brush number, the number of particles on the surface obviously tends to decrease, and the porosity significantly decreases. This decrease is undoubtably a necessary consequence of the increasing gelatin content. After calcination, compared with the corresponding uncalcined sample, the porosity between grains for the calcined samples obviously increases, which is probably attributed to the thermal decomposition of gelatine in the painting layer. This conjecture is verified by carbon elemental mapping (see Fig. 10). Before calcination, the density of carbon on the surface of the sample increases with the brushed number, but the density of carbon for the corresponding samples clearly decreases after calcination. The above results show that it is feasible to form a pore structure on the surface of the painting layer containing the pigments and binders by calcination.

To verify the hypothesis that the elimination of pores can restore the colour blurring caused by the pores, according to Eq. (1), it is feasible that any substance with a colourless refractive index close to the binder is introduced into the pores. Combined with the above requirements and considering the effectiveness of a liquid in eliminating the interface between particles, a colourless liquid, with a refractive index close to gelatine (1.53), [BMIm]PF_6_ (n = 1.55), was selected here. Figure 9a_3_,b_3_ shows the SEM images of the calcined samples (corresponding to Fig. 9a_2_,b_2_) after the introduction of the ionic liquid. As expected, the pores on the surface of the calcined sample significantly decrease when the calcined samples are filled with the ionic liquid. Notably, the ionic liquid used here is only an example to restore the blurred colour of the painting layer caused by the pores, and the function of the ionic liquid here is just a physical effect.

### The surface chemical composition of the simulated wall paintings

To obtain some information regarding the change in the chemical component on the surface of the simulated wall paintings before and after treatment, some typical elements representing materials used were detected by elemental mapping. The typical elemental mapping for the imitated wall paintings, the calcined simulated wall paintings and the resultant samples treated with the ionic liquid ([BMIm]PF_6_) are shown in Fig. 10. For the elemental mapping, carbon as a typical element was selected to determine the change in the gelatine before and after calcination, and fluorine was selected as a typical element to detect the distribution of the ionic liquid introduced into the calcined wall painting. The carbon density increased with the number of brushed painting solutions containing gelatine and red ochre (see Fig. 10 a_1_,b_1_). However, because of the thermal decomposition of gelatine in the painting layer, the carbon density of the calcined simulated wall paintings dramatically decreases (see Fig. 10a_2_,b_2_). The surface distribution of the fluorine for the corresponding calcined simulated wall paintings treated by the ionic liquid is presented in Fig. 10a_3_,b_3_. The results indicate that the density of the fluorine evidently increases with the number of the brushed ionic liquids. The results combined with the SEM images of the corresponding samples in Fig. 9 indicate that the ionic liquid could be effectively introduced into the pores of the calcined samples.

The results from the effects of calcination on the surface morphology and the mixture of red calcined ochre and gelatine definitely show that employing calcination to construct the model of the painting layer with degraded gelatine and unchanged red ochre is feasible and the non-volatile ionic liquid could effectively fill pores to eliminate the interface between the grains on the surface of the painting layer.

### Colour of the simulated wall paintings

**Figure 11:**
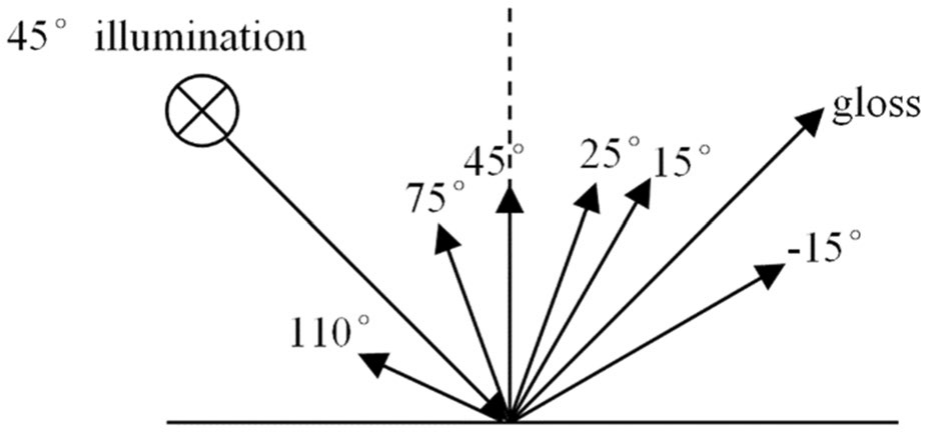
Sketch of medium illumination in-plane detection, MA98.

**Figure 12:**
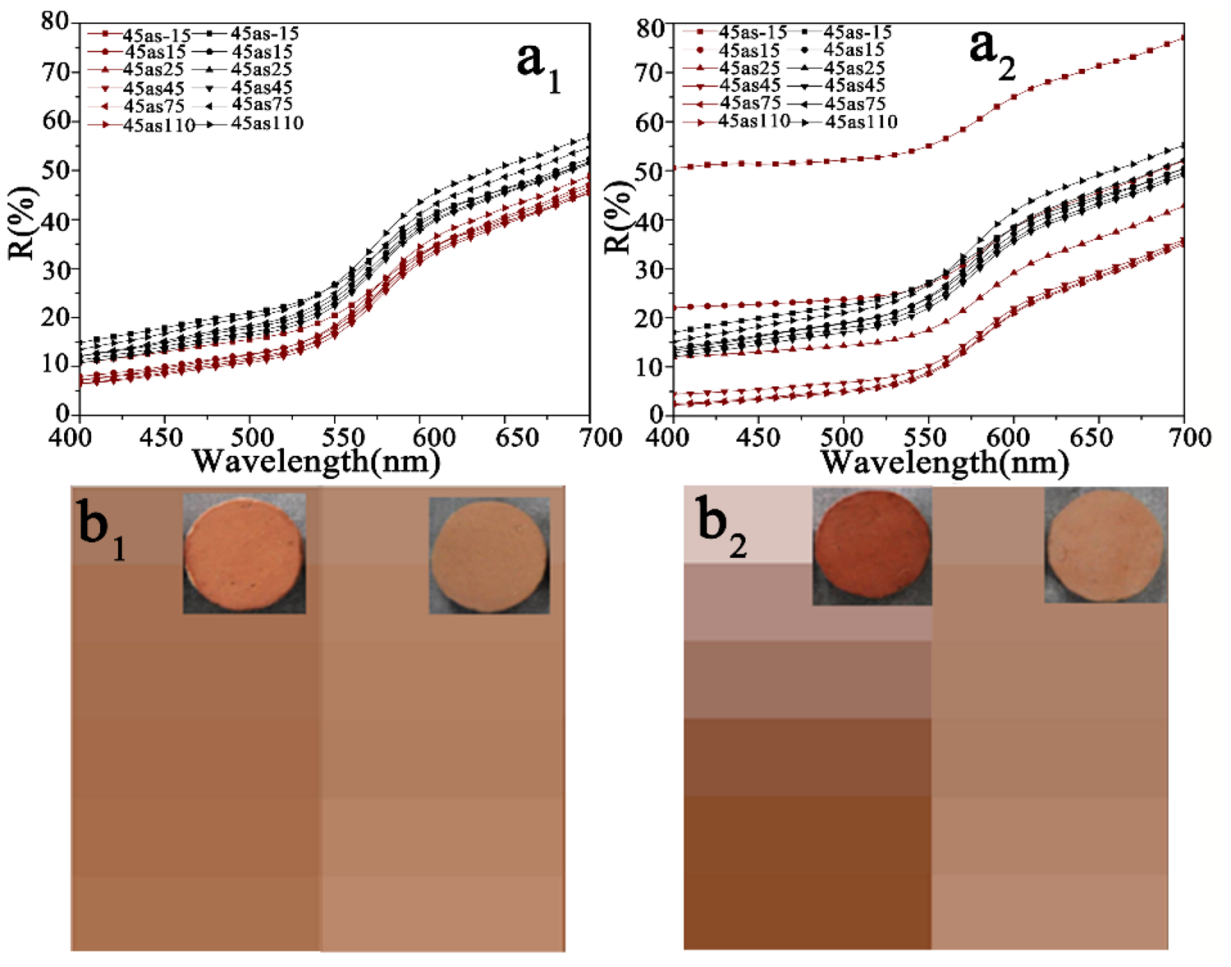
Multiangle reflectance spectra of **(a**_**1**_**)** 2S (red curves) and F-2S (black curves), and **(b**_**1**_**)** 4S (red curves) and F-4S (black curves). **(b**_**1**_**,b**_**2**_**)** Colour images for different viewing directions corresponding to (**a**_**1**_**)** and (**a**_**2**_**)** (In **b**_**1**_ and **b**_**2**_, images of 2S and 4S are on the left side and images of F-2S and F-4S are on the right side). The insets in **(b**_**1**_**)** and **(b**_**2**_**)** are the photographs corresponding to 2S, F-2S, 4S, and F-4S.

The multiangle reflectance method is a powerful tool for providing some scattering information. This technique provides information on the reflective spectrum over different angles and chroma, hue and brightness value to describe the attributes of any colour, or the difference between a sample and a standard^32^. Because the multiangle reflectance spectrum provides abundant optical information, it is an imperative characterization method for the colour used in many aspects^33–35^. A schematic drawing of the angle distribution of the reflective light detected by MA98 showed in Fig. 11. The region located to the right of 45° is more sensitive to the specular reflection related to the smoothness, and the region located to the left of 45° is more sensitive to the diffuse reflection related to the roughness^36^. Employing this method, the multiangle reflectance spectra for the simulated wall paintings were recorded,

Figure 12a_1_, a_2_ show the multiangle reflectance spectra of the simulated wall paintings (corresponding to samples 2S and 4S, red curves) and the calcined samples (corresponding to samples F-2S and F-4S, black curves), and the colour attributes of L*, C*, h* values for the corresponding samples are shown in Table 1. From these data, the following results could be found.

First, for both the simulated wall paintings and the corresponding calcined samples, the profiles of reflective spectra are very similar, implying that these samples have similar hues, and the relatively higher reflectivity at wavelengths ranging from 580 to 700 nm indicates that these samples exhibit a red hue^37–39^. These results implied that the calcination did not alter the hue of simulated wall paintings. Namely, the calcination did not have a significant effect on the light absorption spectral band of red ochre.

Second, for given simulated wall painting and corresponding calcined sample, there are significant differences in the brightness (L*) and saturation (C*) values between them. As shown in Fig. 12, regardless of the 2S or 4S samples, the reflectance spectra of their calcined samples (F-2S, F-4S, black curves) are almost the same, while the reflectance intensity ranging from 400 to 700 nm increases (red curves) compared with their uncalcined samples. (The particularity of the spectra at -15^°^ and 15° in Fig. 12a2 is elaborated in the following paragraph). These results demonstrate that the decomposition of gelatine could make the reflectance intensities in the visible region elevate, implying the increase in brightness (see L* in Table 1)^41^. Combined with the results shown in Fig. 9, concerning the influence of calcination on the surface morphology of simulated wall paintings, the increase in brightness of calcined samples is attributed to the increase in roughness due to the decomposition of gelatine. This conclusion is consistent with the literature reported that the scattering caused by the rough surface morphology hides the appearance of pigment color^40^. Namely, the rough surface could blur the color of the wall painting.

Third, in terms of the saturation, the saturation (C*) value for a given simulated wall painting is higher than that of the corresponding calcined sample. The decrease in saturation here is probably related to the white residue of the calcined gelatin^42^. As stated earlier, there are certain amounts of inorganic minerals, such as Ca(CO_3_)_2_, phosphate, and aluminosilicate in the calcined gelatin residue.

Finally, there is a difference in the colour attributes between the simulated wall paintings with different brushed numbers, which is mainly related to the difference in the amount of gelatin. For example, for the 4S sample, due to too much gelatine, the gloss effect leads to obviously higher reflective spectra at – 15° and 15° (located at the sensitive monitoring region of the specular reflection) in the whole wavelength range as shown in Fig. 12a2.

Based on the results demonstrated above, it could conclude that the degradation of gelatine in the painting layer through calcination result in an increases in the roughness, which in turn enhances scattering to cause colour blurring.

### Restoration of the blurred colour of the calcined simulated wall paintings

**Figure 13:**
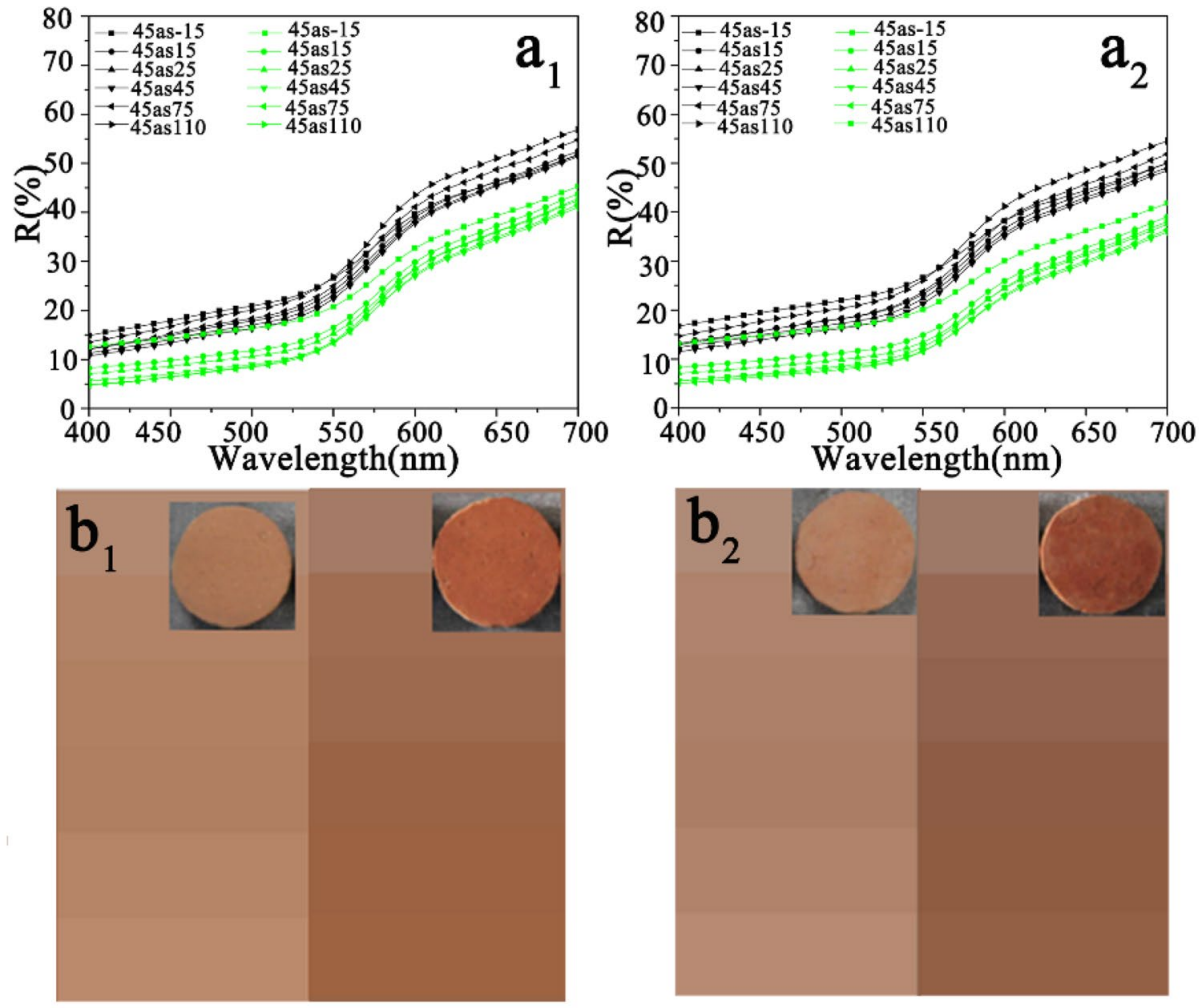
Multiangle reflectance spectra of **(a**_**1**_**)** F-2S (black curves) and CF-2S (green curves) and **(b**_**1**_**)** F-4S (black curves) and CF-4S (green curves). **(b**_**1**_**,b**_**2**_**)** Colour images at different viewing directions corresponding to **(a**_**1**_**)** and **(a**_**2**_**)** (In **b**_**1**_ and **b**_**2**_, images of F-2S and F-4S are on the left side and images of CF-2S and CF-4S are on the right side.). The insets in **(b**_**1**_**)** and **(b**_**2**_**)** are the photographs corresponding to 2S, F-2S, 4S, and F-4S.

To evaluate the performance of the ionic liquids for the restoration of the blurred colour of the calcined simulated wall paintings, the multiangle reflectance spectra of calcined simulated wall paintings before and after the introduction of the ionic liquid were recorded. The results are shown in Fig. 13. Clearly, the profiles of the reflective spectra of CF-2S and CF-4S are similar to those of F-2S and F-4S, respectively, showing that the introduction of the ionic liquid into the calcined simulated wall paintings hardly affects the colour hue (see h* in Table 1). However, the reflective spectra of CF-2S and CF-4S clearly move down more than those of F-2S and F-4S, almost close to the original level of the corresponding simulated wall paintings (see Figs. 12 and 13, respectively). The photographs of the corresponding samples shown in Figs. 12 and 13 directly reflect the changes mentioned above. Some detailed information about the colour attributes shows in Table 1. In terms of the brightness, the brightness (L*) value of a given calcined simulated wall painting is higher than that of the calcined sample after the introduction of the ionic liquid, which could be attributed to a decrease in the surface roughness due to the filling of ionic liquid into the pores, as shown in Fig. 9. It is worth noting that the introduction of ionic liquids did not restore L* and C* to the extent that of the original simulated wall painting. These differences may be related to the higher refractive index of the ionic liquid than gelatine. A medium with a higher refractive index could lead to incident light traversing a good distance inside the solid and can become strongly coloured^40^. Additionally, compared with the dried gelatine in the painting layer, the ionic liquid has a strong permeability, which means that it has a strong ability to eliminate the interfaces between the grains in the painting layer. As a result, the ionic liquid has a strong ability to eliminate light scattering than gelatine.

Although the above approach cannot achieve the perfect restoration of the blurred colour of the simulated wall painting, it provides a new strategy for developing blurred wall paintings in principle. Regardless of which materials are desirably selected to fill the surface pores of the wall paintings, the refractive index needs to be close to that of the original binder and requires a strong compatibility with the wall pores in the painting layer. Additionally, to ensure the restored colour effect, the amount of materials introduced into the blurred wall painting should be as small as possible.

### Restoration of a blurred ancient wall painting

**Figure 14:**
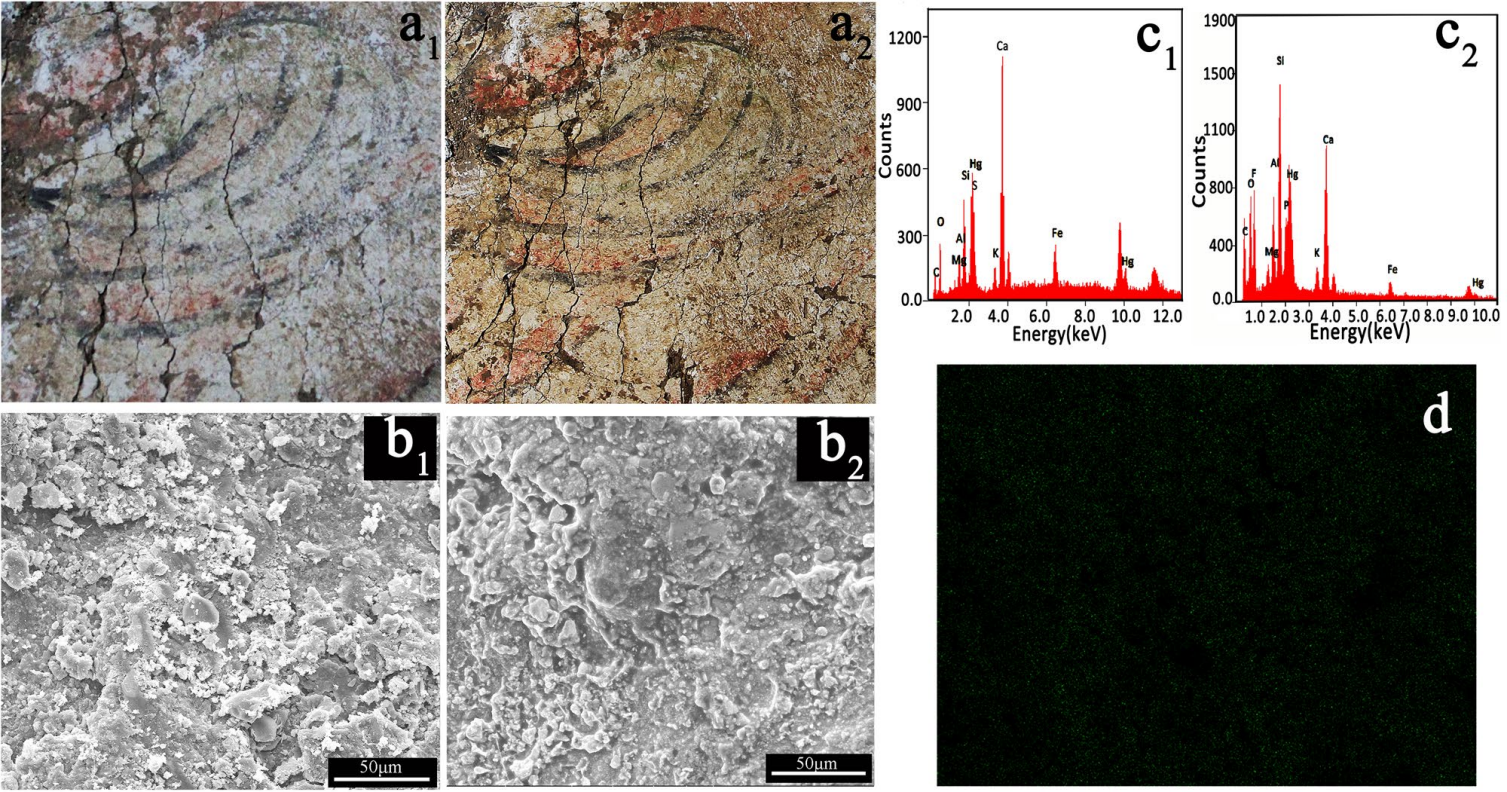
Photographs of the partial ancient mural (Tang Dynasty, Sanyuan, Shananxi, China) before **(a**_**1**_**)** and after **(a**_**2**_**)** treatment with the ionic liquid. SEM images (**b**_**1**_,**b**_**2**_) corresponding to the sample in **(a**_**1**_**)** and the sample in **(a**_**2**_**)**. **(c**_**1**_**,c**_**2**_**)** EDS spectra corresponding to the sample in **(b**_**1**_**)** and **(b**_**2**_**)**. **(d)** F element mapping corresponding to **(b**_**2**_**)**.

To illustrate the practicability of the principle we proposed in this investigation, a fragment of the blurred ancient tomb wall painting (Zang Huailiang Tomb, AD 730, Tang Dynasty, Sanyuan, Shaanxi, China) was restored as an example, and the results are presented in Fig. 14. As shown in Fig. 14a_1_, the colour on the wall painting is already blurred. However, as expected, the blurred red colour is remarkably restored after treatment with an acetone solution containing the ionic liquid (see Fig. 14a_2_). The SEM images of the ancient wall painting and after treatment with the ionic liquid are given in Fig. 14b_1_, b_2_. Before the treatment, the painting layer exhibits a rough surface, with a large number of pores formed between grains (see Fig. 14b_1_). After the ionic liquid treatment, the surface becomes smooth because the pores are filled with the ionic liquid (see Fig. 14b_2_). The filling effect of the ionic liquid could be confirmed by the EDS spectra and fluorine element mapping, as shown in Fig. 14c_1_,c_2_. Before treatment by using the ionic liquid, the characteristic peaks related to C, O, S, Mg, Al, Si, K, Ca, Hg and Fe appear (see Fig. 14c_1_). The presence of C, O, S, Mg, Al, Si, K and Ca probably comes from the components of clay and lime^43^. Nevertheless, the obvious Fe, S, and Hg in the spectrum released by the red pigment is possibly cinnabar or red ochre. Although the red pigment exists in the blurred wall painting, the red colour is not effectively present. After the ionic liquid treatment (see Fig. 14c_2_), the signal from F obviously appears, and the uniform distribution of F displayed in Fig. 14d also confirms this. These results show that the surface of the treated painting is evenly covered by the ionic liquid, which restored the red colour and the black colour of the blurred wall painting.

**Figure 15:**
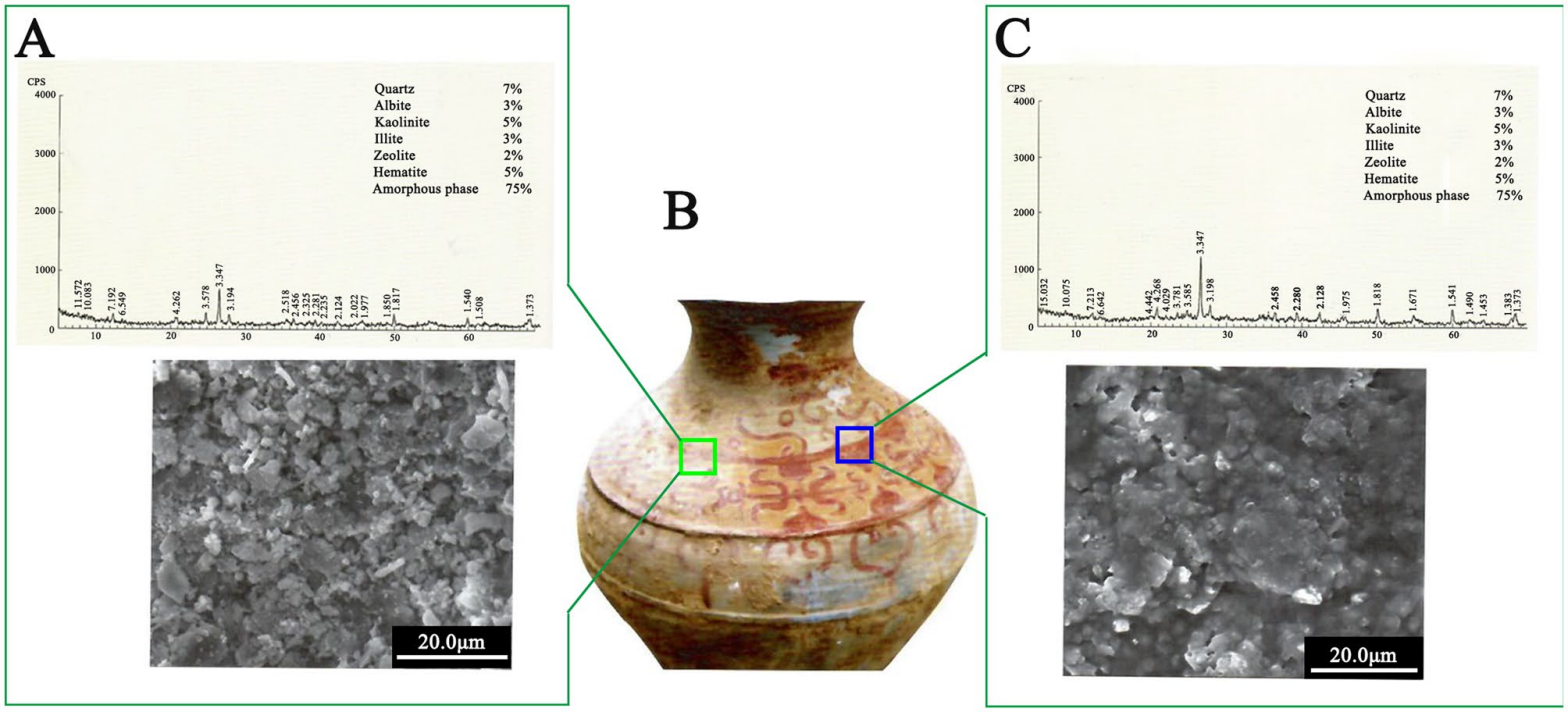
**(A)** XDR spectrum (top) and SEM image (underneath) of blurring pigment layer corresponding to the site of the green square in photograph B. **(B)** Photograph of colored pottery (Eastern Han Dynasty, Shaanxi, China), **(C)** XDR spectrum (top) and SEM image (underneath) of the blurred pigment layer after ionic liquid treatment corresponding to the site of the blue square in photograph **(B)**.

To demonstrate the effectiveness of our proposed method in developing blurred coloured drawings, a pottery jar with a blurred red painting from the Eastern Han Dynasty) was selected for a similar treatment. Although red pigment haematite exists in the blurred area (see left XRD spectrum in Fig. 15), its colour was blurred, the SEM image shows that the surface of this area was obviously rough (see left SEM image in Fig. 15). For the area restored, decorative patterns appeared remarkably, and the surface of the treated area became smooth (see right SEM image in Fig. 15). The corresponding XRD spectrum indicates that the components are almost same as that of the blurred area. The clear color of the treated area of the pottery jar has not changed significantly since restoration in 2009. This example shows not only the reliability of the principle of the method in practical application, but also the durability of restoring the color to some extent.

## Conclusions

In ancient China, the so-called *secco* technique has been popularly used. For this kind of wall painting, the pigments are bound in any film-forming binder, such as animal glue or vegetable glue. Universally, the blurring of ancient Chinese wall paintings is often considered to be caused only by the changes in pigments. In this paper, the effect of binder decay on wall painting blurring was investigated. Based on the simulated experimental results, the following conclusions could be drawn. (1) Binder decay can cause the blurring of the wall paintings even if the pigments do not change. The decay of the binder as continuous phase in the painting layer leads to the formation of dispersed pores, and the surface light scattering originating from these pores makes the brightness of the wall painting increase. As a result, the colour of the wall painting becomes blurry. (2) Introduction of a colourless and non-volatile ionic liquid with a refractive index close to the binder into the pores formed by the decay of the binder in the wall paintings could restore the blurred colour to some extent, and the main reason is the decrease in the brightness of the wall painting caused by scattering. (3) Although the complexity of the interaction between the materials in the painting layer makes it almost impossible to perfectly restore the blurred colour of the wall painting by our proposed approach, the elimination of light scattering has no fundamental effect on the hue of the colour. (4) In principle, the approach proposed in this study is universal, and it is suitable for the restoration of various kinds of blurred paintings not caused by the decay of pigments or dyes. We believe that the mechanism proposed in the present paper is significant for researching cultural heritage.

## Methods

### Materials

Ochre pigment (particle size in 400 mesh), gelatine (Tianjin Chemical Reagent Co., Ltd, Tianjin, China), 1-butyl-3-methylimidazolium hexafluorophate ([BMIm]PF_6_, Zhongkekaite Industry and Trade Co., Ltd, Lanzhou, China), Gelatine and acetone (Sinopharm Chemical Reagent Co., Ltd, Shanghai, China) were used as received without further purification. Red ochre pigment was purchased from Anhui Highkey Import & Export Co., Ltd, which was calcined in Muffle furnace at 650 °C for 2 h before use. Water (resistance, 18 MΩ.cm) was used in all preparations of the solutions.

### Preparation of solutions

The concentration of the gelatine solution was the mass ratio of gelatine to water (30:100). The pigment solution was prepared by evenly dispersing 0.061 g calcined red ochre powder (red ochre calcined at 650 °C for 2 h) into 5 mL of the above gelatine solution. An acetone solution of ionic liquid ([BMIm]PF_6_) was prepared at a volume ratio of [BMIm]PF6 to acetone of 1:2.

### Preparation of the simulated wall paintings with porous scattering

The simulated wall paintings with porous scattering was as follows. The cylindrical compressed soil samples (3 cm in diameter × 1 cm in height) were first prepared by pressing a mixture of 50 g soil, 5 g flour, and 20 mL water in the cylinder mold and then were dried in air. The dried soil samples were calcined at 650 °C for 2 h in a muffle furnace. The surfaces of the calcined soil samples were polished by a fine grade of sandpaper, and the base materials for the simulated wall paintings were finally obtained.

To obtain the simulated wall paintings with different colour identities, the base materials were brushed at various times via the calcined red ochre pigment solution. The samples brushed two and four times are denoted as 2S and 4S, respectively. These two samples were calcined in the muffle furnace at 650 °C for 2 h. Due to the thermal decomposition of different amounts of gelatine in these pigment layers, the calcined samples become wall paintings with various porous scattering, which denoted as F-2S, and F-4S, respectively.

### Restoration of the simulated blurred wall paintings

To fill the pores in the simulated blurred wall paintings, these samples were penetrated with 200 µL of acetone solution containing the ionic liquid. After the evaporation of acetone, the restored simulated blurred wall paintings were obtained.

### Restoration of the blurred ancient mural painting

A piece of the fragment taken from the wall painting of the Sanyuan Zanghuailiang tomb (Tang Dynasty, ~ 730, Shaanxi, China) and a pottery jar with a blurred red painting from the Eastern Han Dynasty were treated to restore the blurred colour. The detailed restoration process is as mentioned above.

### Characterizations

#### Composition characterization

FTIR spectra were recorded on KBr discs using a Fourier transform infrared spectrometer (Tensor27, Bruker, German) in transmission mode, and the spectra were collected from the range of 4000 cm^−1^ to 400 cm^−1^ with a typical resolution of ± 4 cm^−1^.

The crystal structures of ochre before and after calcination were determined using an X-ray diffractometer (DX-2700, Dandong Haoyuan, China). The diffractometer wasoperated at 40 kV and 30 mA with Cu Kα irradiation (λ = 0.15418 nm), and the detection range was from 10° to 80°.

X-ray Photoelectron Spectroscopy (Axis Ultra, Kratos Analytical Ltd.) was used to perform XPS with achromatic Al Kα radiation (1486.6 keV). High-resolution scans were performed for carbon (C 1s) and iron (Fe 2p). CasaXPS software was used for the XPS data analysis. All binding energies were calibrated using the C 1s peak at a fixed value of 284.8 eV.

Thermal analysis of gelatine was detected by using a thermo-analyser system (Q1000DSC + LNCS + FACS Q600SDT, USA) under an air atmosphere. The range of heating was from room temperature to 800 °C, and the heating rate was 10 °C/min.

### Morphological observation

All SEM images, EDS spectra, and elemental mapping data were measured via a scanning electron microscope (Quanta 200, FEI, Netherlands) operated at 20.0 kV associated with energy dispersive X-ray spectroscopy (EDS) under high vacuum mode.

### Multireflective spectroscopy

Multireflective spectra at various viewing directions were recorded in the range of 400–700 nm by a multiangle spectrophotometer (MA98, X-Rite, USA) with standard illumination of D65, and the corresponding colour images and L^*^A^*^B^*^ values were also obtained.
